# Subconjunctival dexamethasone implant for non-necrotizing scleritis

**DOI:** 10.1186/1869-5760-3-7

**Published:** 2013-01-07

**Authors:** Heloisa Nascimento, Maíra França, Luciana Guadalupe García, Cristina Muccioli, Rubens Belfort

**Affiliations:** 1 Department of Ophthalmology, Paulista School of Medicine, Hospital São Paulo, Federal University of São Paulo, São Paulo, Brazil; 2 Department of Ophthalmology, Universidad de Córdoba, Córdoba, Argentina

## Abstract

**Background:**

The purpose of this study is to report the management of non-necrotizing anterior scleritis with a single-dose subconjunctival 0.7 mg dexamethasone implant (Ozurdex®, Allergan, Inc., CA, USA). Six patients with clinical diagnosis of non-necrotizing anterior scleritis (diffuse, sectorial, and nodular) were submitted to subconjunctival injection of dexamethasone implant. The injection was performed under topical anesthesia at the slit lamp. All patients reported only mild discomfort related to the procedure. Five patients had subconjunctival hemorrhage. Follow-up was performed 1, 7, 15, 30, and 45 days, and 2, 3, 4, 5, and 6 months after the procedure. Visual acuity, intraocular pressure, anterior and posterior biomicroscopy, and fundus exams were performed in each visit.

**Results:**

In all patients, symptoms disappeared before day 7, and most of them were symptoms-free on day 2. The implant was visible at least up to day 45. One recurrence was noted in the 6-month follow-up in a patient with rheumatoid arthritis and non-necrotizing diffuse scleritis and was treated with oral steroids. No patient developed ocular hypertension or any kind of complications during the follow-up period, except for subconjunctival hemorrhage.

**Conclusion:**

Dexamethasone implant was safely and effectively used as a local therapy for non-necrotizing scleritis.

## Background

Anterior scleritis is usually a chronic, painful, progressive, potentially blinding condition involving both the episclera and the sclera [1,2]. It is divided in diffuse, nodular, and sectorial scleritis depending on the clinical appearance. It can also be necrotizing or non-necrotizing; infectious or non-infectious. It is often associated with ocular complications (anterior uveitis, peripheral keratitis, and glaucoma), potentially causing decrease of vision, and with systemic connective tissue or vasculitic diseases, some of them potentially lethal [2,3].

The management of scleritis is a challenge. Topical eyedrops frequently are ineffective. Systemic administration of nonsteroidal anti-inflammatory drugs (NSAIDs), corticosteroids, nonsteroidal immunosuppressive agents, or a combination, is the mainstay of treatment for non-infectious scleritis [4]. These therapies can have serious adverse side effects and morbidity. Systemic corticosteroids are often accompanied by a poor safety profile characterized by multiple adverse effects, such as fluid retention, hypertension, hyperglycemia, greater susceptibility to infections, osteoporosis, mood changes, and psychosis [5].

Regional steroid injections have been successfully used in the treatment of scleritis [6,7]. Although classic teaching purports that subconjunctival application of steroids for scleritis is controversial, if not dangerous, because of the alleged risk of scleral necrosis and melt [6], there have been several published series of patients who have received periocular triamcinolone acetonide injection for non-necrotizing, noninfectious anterior scleritis [8].

Regional steroids have been avoided in the treatment of scleritis because of concerns about the risk of scleral thinning or perforation. The literature supporting this notion is comprised of a few case reports. A literature review revealed a few cases of scleral thinning in scleritis patients attributed to subconjunctival steroid injections. However, reports of rare events like scleral melts are circumstantial by nature and preclude an accurate assessment of a true occurrence rate [6]. Sohn et al. reported in a retrospective study of subtenonian triamcinolone injection for anterior scleritis no cases of scleral thinning or necrosis, but long-term follow-up of these small series was lacking [7].

Subconjunctival steroid injections have become part of the legitimate armamentarium for scleritis treatment in the last decade. It may be an attractive adjunct to systemic therapy by achieving timely improvement while systemic medications begin to take effect [6-9].

Dexamethasone (DEX) 0.7 mg implant (Ozurdex®, Allergan, Inc., CA, USA) is a biodegradable implant approved by FDA. It was designed to be injected into the eye (vitreous) to treat adults with macular edema following branch retinal vein occlusion or central retinal vein occlusion. It is also indicated to treat adults with noninfectious uveitis affecting the posterior segment of the eye. This implant is composed of a mix of polylactic acid and polyglycolic acid polymers and can be implanted into the eye using an office-based procedure. As the implant erodes, dexamethasone is released into the eye. The most common side effects reported in patients include the following: increased eye pressure, conjunctival bleeding, eye pain, conjunctival hyperemia, ocular hypertension, cataract, and headache [5].

This implant represents a new approach to the treatment of ocular diseases since it is capable to promote a 3-day local pulse therapy followed by a 6-month gradually sustained release when it is placed in the vitreous. There is no study of its pharmacokinetic in the subconjunctival space. The purpose of this study was to evaluate the safety and efficacy of single-dose off-label subconjunctival slow delivery system of 0.7 mg DEX for recurrent non-necrotizing anterior scleritis.

## Materials and methods

The study was approved by the Institutional Review Board and followed the tenets of the Declaration of Helsinki. Informed consent was obtained from all subjects prior to the study and after an explanation of the nature and possible consequences of the study.

Scleritis was diagnosed on the basis of the characteristic clinical picture of painful inflammation and tenderness that radiated to the forehead, brow, jaw, or sinuses, with edema affecting the episcleral and scleral tissues, and injection of both the superficial and deep episcleral vessels; congestion of the deep episcleral vessels remained after the application of 10% phenylephrine drops. Anterior scleritis was characterized as diffuse, sectorial, or nodular according to the clinical appearance.

Patients with infectious refractory non-necrotizing anterior diffuse, sectorial, or nodular scleritis despite adequate treatment that required steroid therapy were referred for single-dose DEX 0.7 mg subconjunctival slow delivery implant according to investigator judgment.

**Figure 1:**
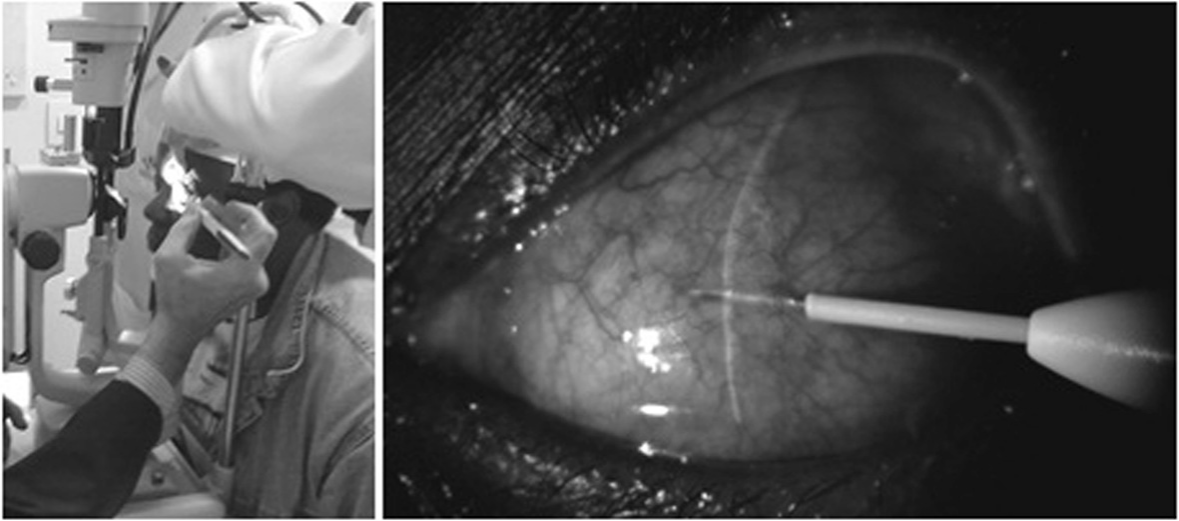
Subconjunctival injection of DEX 0.7 mg implant at the slit lamp.

Single-dose DEX 0.7 mg implant was performed with the patient seated at the slit lamp (Figure 1). Before the procedure, topical anesthesia and 5% iodine-povidine solution were instilled. The same investigator performed all the injections (RB). In nodular and sectorial scleritis, the implant was placed in the area adjacent to the inflammation. In diffuse scleritis cases, the implant was placed at the areas of maximal inflammation. After the procedure, patients received local prophylactic antibiotic regimen for 7 days.

Patients were followed 1, 7, 15, 30, and 45 days, and 2, 3, 4, 5, and 6 months post-procedure. Visual acuity, intraocular pressure (IOP), anterior and posterior biomicroscopy, and fundus exams were performed in all visits.

## Results

Six patients with non-necrotizing anterior nodular, sectorial, or diffuse refractory scleritis were included in this study (Table 1). Four were female and two were male. Mean age was 39.8 years (range 28 to 60 years). Two patients presented scleritis related to rheumatoid arthritis, one probably related to tuberculosis, and three were idiopathic. Tuberculin skin test was the only positive finding in the case probably related to tuberculosis. All patients were on stable systemic or local therapy, and no increase to their therapy was made prior to enrolment in this study. In all patients, symptoms disappeared before day 7, and most of them were symptoms-free on day 2. The implant was visible at least up to day 45 in all patients. One recurrence was noted in the 6-month follow-up in a patient with rheumatoid arthritis and diffuse scleritis, and was treated with oral steroids. No patient developed ocular hypertension or any kind of complications during the follow-up period, except for subconjunctival hemorrhage in five patients which resolved spontaneously.

**Table 1:** Patients' data summary

**Patient**	**Age (years)**	**Gender**	**Scleritis type (all anterior non-necrotizing)**	**Etiology**	**Concomitant medications**	**Recurrence**
1	45	Female	Sectorial	Rheumatoid arthritis	Indomethacin (150 mg/day), methotrexate (25 mg/week), etanercept (50 mg/week SC), prednisone (1 mg/kg/day)	One recurrence on the 6th follow-up month
2	32	Male	Diffuse	Idiopathic	Indomethacin (150 mg/day), Prednisone (1 mg/kg/day)	-
3	60	Female	Nodular	Rheumatoid arthritis	Diclofenac (150 mg/day), etanercept (50 mg/week SC), methotrexate (25 mg/week), prednisone (1 mg/kg/day)	-
4	28	Male	Diffuse	Idiopathic	Prednisone (1 mg/kg/day)	-
5	32	Female	Nodular	Tuberculosis	Isoniazid (400 mg/day), rifampicin (600 mg/day), pyrazinamide (2,000 mg/day), ethambutol (1,200 mg/day)	-
6	42	Female	Diffuse	Idiopathic	Indomethacin (150 mg/day), prednisone (1 mg/kg/day)	-

SC, subconjunctival.

## Discussion

The management of scleritis is challenging in ophthalmology. Systemic administration of NSAIDs, corticosteroids, immunosuppressive agents, or a combination, is many times required for long time [1-4]. Although it is considered the mainstay of treatment for non-infectious scleritis, compliance, and side effects preclude effective treatment [5-7].

Nonsteroidal anti-inflammatory drugs can lead to gastrointestinal issues including ulcers and gastritis. Steroidal drugs can worsen systemic conditions as diabetes and arterial hypertension.

Systemic immunosuppressive therapy is effectively used for ocular inflammatory diseases as corticosteroid-sparing therapy for corticosteroid-responsive, corticosteroid-dependent disease in order to avoid long-term use of high doses of oral corticosteroids, as supplemental therapy when systemic corticosteroids fail to suppress ocular inflammation, and for specific diseases, such as Behçet’s disease, in which early use of immunosuppressive therapy is thought to confer better outcomes [4].

Immunosuppressive agents may have significant side effects and may not be safe for individuals with comorbidities and those who are pregnant. Also, it can increase the risk of malignancies [10-12]. Developing countries like Brazil does not have the availability of such drugs for ocular treatment.

The Systemic Immunosuppressive Therapy for Eye Diseases study has recently shown data regarding the overall mortality or cancer mortality after treatment with biological response modifiers (BRM) for inflammatory diseases [12,13]. Because of these reasons, BRM agents are not first-line therapy for scleritis.

In rheumatoid arthritis advanced cases, evidence has emerged that the initiation of nonbiologic disease-modifying antirheumatic drugs (DMARDs), including methotrexate (MTX), early in the course of rheumatoid arthritis clearly has a major impact on the progression of disability [10,11]. However, despite the use of adequate DMARDs, the first patient submitted to subconjunctival single-dose DEX 0.7 mg implant (Ozurdex®, Allergan, Inc.) still presented scleritis activity even with controlled arthritis activity. Treatment options were exhausted for her. Local subconjunctival dexamethasone implant was an effective approach to control her non-necrotizing scleritis for at least 6 months and represents a new option for refractory cases.

Ozurdex® (Allergan, Inc.) has already obtained FDA approval for intravitreal use for macular edema due to non-infectious uveitis or retinal vein occlusions [5]. It had never been used before for scleritis and subconjunctivally. Although patients of this study had been refractory to the treatment of scleritis, only one recurrence was noted in the 6-month follow-up, which suggests that steroid slow release directly to the sclera can be crucial in scleritis physiopathology and management. However, pharmacodynamics of the implant has not been tested in the subconjunctival space.

Three out of the six patients included in this trial had the diagnosis of idiopathic unilateral recurrent non-necrotizing anterior scleritis. In these cases, one advantage of controlling scleritis locally is not to expose the patient to the risks of systemic immunosuppression. Also, signs and symptoms of the potential causative disease would not be masked by the systemic immunosuppression, possibly allowing the proper diagnosis and management of the potential underlying disease.

There is also a general concern about IOP increase with the use of local steroids. However, it was not noted in any scleritis patient treated with subconjunctival dexamethasone implant. More studies need to be performed to better elucidate this issue.

Although not noted in this study population, disadvantages of subconjunctival dexamethasone implant can include increased IOP and scleral melt. In this case, it could be easier to remove the implant differently from what occurs with triamcinolone acetonide injections. Another great problem of the implant is its high cost, which can preclude its use. Further studies comparing subconjunctival dexamethasone implant and triamcinolone injections should be performed to better evaluate those issues.

## Conclusion

Single-dose DEX 0.7 mg implant was safely and effectively used for the local treatment of non-necrotizing anterior scleritis. Potential advantages could include easier steroid removal in case of complications such as scleral melting or glaucoma. Also, it would not mask systemic diseases, signs, and symptoms, allowing proper diagnosis of the scleritis cause. Cost-effective relationship should be assessed.

## Competing interests

The authors declare that they have no competing interests.

## Authors' contributions

HMN, MF, LGG data collection; HMN: writing the manuscript; RBJ, CM: manuscript revision; RBJ: final revision. All authors read and approved the final manuscript.

## Authors' information

MHK is a PhD student in Material Science. MM is a PhD student in Chemical Engineering. MK is an associate professor of Chemical Engineering. SRK is a PhD student in Material Science.
